# Lnc_000048 Promotes Histone H3K4 Methylation of MAP2K2 to Reduce Plaque Stability by Recruiting KDM1A in Carotid Atherosclerosis

**DOI:** 10.1007/s12035-023-03214-0

**Published:** 2023-01-23

**Authors:** Shuai Zhang, Yu Sun, Qi Xiao, Mengying Niu, Xudong Pan, Xiaoyan Zhu

**Affiliations:** 1 Department of Neurology, The Affiliated Hospital of Qingdao University, Qingdao, China; 2 Department of Critical Care Medicine, Shandong Provincial Hospital Affiliated to Shandong First Medical University, Jinan, China; 3 Department of Critical Care Medicine, The Affiliated Hospital of Qingdao University, Qingdao, China

**Keywords:** Long non-coding RNA, Atherosclerosis, KDM1A, MAP2K2

## Abstract

Stabilizing and inhibiting plaque formation is a key challenge for preventing and treating ischemic stroke. KDM1A-mediated histone modifications, which involved in the development of training immunity, ultimately exacerbate the outcomes of inflammation. Although lncRNAs can recruit KDM1A to participate in histone methylation modification and regulate inflammation, cell proliferation, and other biological processes, little is known about the role of KDM1A-lncRNA interaction during atherosclerosis. The present study sought to delineate the effect of the interaction between lnc_000048 and KDM1A on plaque rupture in carotid atherosclerosis, as well as the potential mechanism. Our results revealed that lnc_000048 reduced the activity of histone demethylase and activated MAP2K2 expression by interacting with KDM1A. Furthermore, upregulated lnc_000048 indirectly regulated ERK phosphorylation by MAP2K2 and eventually activated the inflammatory response through the MAPK pathway, which was involved in atherosclerosis. Importantly, our study using ApoE-/- mice confirmed the regulatory role of lnc_000048 in promoting inflammation and collagen degradation in atherosclerotic plaques. These results suggest that targeting the lnc_000048 /KDM1A/MAP2K2/ERK axis may be a promising strategy for preventing atherosclerosis.

**Supplementary Information:**

The online version contains supplementary material available at 10.1007/s12035-023-03214-0.

## Background

Atherosclerosis-induced unstable plaque plays a crucial role in ischemic stroke [1, 2]. Understanding the pathophysiological process and regulatory mechanism of atherosclerosis is of strategic significance for preventing and treating ischemic stroke. Aggravated inflammation and increased expression levels of matrix metalloproteinases (MMPs) can weaken plaque caps and promote plaque rupture [3, 4]. Further, lncRNA-mediated histone methylation plays an important role in chronic inflammatory vascular diseases, such as atherosclerosis [5–7].

LncRNAs contribute to atherosclerotic processes such as lipid metabolism disorders, inflammatory responses, and plaque formation [8]. Leisegang et al. found that the lncRNA MANTIS limited ICAM-1 mediated monocyte adhesion to endothelial cells, which may accelerate the development of atherosclerosis [9]. LncRNAs can also participate in histone modification by binding proteins and interfering with the transcription of inflammatory factors, chemokines, and metabolic pathways [10, 11]. KDM1A (also known as LSD1) plays an important pathological role by regulating histone methylation modification (H3K4me1/2 and H3K9me1/2), which leads to epigenetic reprogramming and affects gene expression [12, 13]. Choi et al. reported that the lncRNA MEF2 recruited histone demethylase KDM1A to decrease the levels of inhibitory markers such as H3K9me2 and H3K9me3 from the promoter region of muscle-specific genes and subsequently promoted the differentiation of muscle cells [14]. The binding of lncRNAs to KDM1A is becoming an important target for disease treatment as it leads to reversible changes in gene transcription caused by histone modification [15–17]. However, the specific mechanisms by which lncRNAs interact with KDM1A during atherosclerosis initiation and development need to be further investigated.

Our earlier research showed that the lnc_000048 was highly expressed in plasma exosomes of patients with large-artery atherosclerotic stroke, and the elevation of its level was related to the rupture of atherosclerotic plaques [18]. But it is still unknown how lnc_000048 contributes to atherosclerosis. We hypothesized that lnc_000048 could interact with KDM1A in target genes’ promoter regions and thus involved in atherosclerosis. We designed this study to explore the potential impact of histone modification induced by the interaction between lnc_000048 and KDM1A on plaque formation and stability in atherosclerosis, with the aim of identifying a new potential molecular mechanism underlying atherosclerosis progression.

## Methods

### Bioinformatic Analysis

Lnc_000048 was identified from the RNA sequencing [18]. In the Supplementary Materials and Methods, a comprehensive bioinformatic analysis was detailed.

### Cell Culture and Drug Treatment

Procell Life Science&Technology Co., Ltd. provided the human mononuclear cell line (THP-1, Procell Life Science&Technology, CL-0233) for purchase (Wuhan, China). The Supplementary Materials and Methods described the specifics of cell culture. THP-1 cells were cultured in 6-well plates or 10 cm^2^ dishes as required for the exponential phase. The cells were then given a 100 nM PMA (Sigma-Aldrich Chemical Company, USA) treatment for 48 h to induce monocytes into macrophages. Next, 100 μg/mL ox-LDL (Yiyuan Biotechnology, Guangzhou, China) was added to serum-free RPMI 1640 medium for 48 h to construct the atherosclerosis model in vitro for subsequent experiments. According to the experimental requirements, the model cells were pretreated with signaling pathway inhibitors or enzyme inhibitors, including MEK/ERK inhibitors (FR 180,204, terminal concentration 1 µM) and GSK-LSD1 (final concentration 5 µM) [19, 20]. All inhibitors were purchased from MCE. Lentivirus was transfected into THP-1 cells in accordance with the manufacturer’s recommendations. The Supplementary Materials and Methods provided a description of the specifics.

### Carotid Atherosclerosis Model and Transfection in ApoE-/- mice

All experimental animal protocols were approved by the Animal Management Committee and Animal Ethics and Welfare Committee of the Affiliated Hospital of Qingdao University. The US National Institutes of Health’s Guide for the Care and Use of Laboratory Animals was strictly followed when carrying out the protocols (NIH Publication No. 85–23, revised 1996). The model of carotid atherosclerosis was constructed as explained previously [21, 22]. Then, as previously mentioned, the virus-infected cells were injected into the mice’s tail vein for a total of 4 weeks [23–25].

### RNA Isolation and Quantification

RNA isolation and quantification were carried out as previously described [26]. The Supplementary Materials went into detail about these specifics and Methods and primers used for the genes of interest are listed in Supplementary Table S1.

### Protein Extraction and Western Blotting Analysis

Western blotting was carried out in the manner previously described [27]. The Supplementary Materials and Methods provided a description of the specifics.

### Oil Red O Staining

To assess the accumulation of lipid in cells, the Oil Red O Stain Kit was performed as per manufacturer’s instructions (G1262; Solarbio; Beijing, China). The Supplementary Materials and Methods contained further information.

### RNA Pull-Down Assay and Mass Spectrometry Analysis

Using the PierceTM Magnetic RNA–Protein Pull-Down Kit’s instructions, RNA pull-down assays were used to look at the lnc_000048-binding proteins (Thermo Scientific). Details are provided in the Supplementary Materials and Methods and primer sequences of lnc_000048 for RNA pull-down are listed in Supplementary Table S2.

### Fluorescence In Situ Hybridization (FISH)

Fluorescence in situ hybridization assays were performed as per manufacturer’s instructions (C10910; RiboBio; Guangzhou) [28]. FISH was used to assess lnc_000048 distribution in THP-1 cells. Details were provided in the Supplementary Materials and Methods.

### Immunofluorescence

Immunofluorescence was used to assess the distribution of KDM1A in THP-1 cells. The details were described in the Supplementary Materials and Methods.

### Chromatin Immunoprecipitation (ChIP)

To assess H3K4me2, Chromatin immunoprecipitation (ChIP) assays were carried out in accordance with the manufacturer’s instructions (26,156; Thermo Scientific) [29]. The details were described in the Supplementary Materials and Methods and primers used for the genes of interest are listed in Supplementary Table S3.

### HE Staining

HE staining was performed as described previously [30]. The Supplementary Materials and Methods provide details.

### Masson Staining

Masson staining was conducted using a ready-to-use kit (Masson’s Trichrome Stain Kit, Solarbio) [31]. The Supplementary Materials and Methods provide details.

### Statistical Analysis

Data represent the mean ± standard error. Comparisons between the controls and treatment groups were performed using one-way ANOVA. The Mann–Whitney *U* test or Student’s *t*-test was used to compare continuous variables between patients and controls. Statistical significance was defined as *p* < 0.05 for all tests. Statistical analyses were performed using Statistical Package for the Social Sciences software version 17.0 (SPSS Inc., Chicago, IL, USA) and GraphPad Prism 6.

## Results

### Characteristics of lnc_000048

**Fig. 1:**
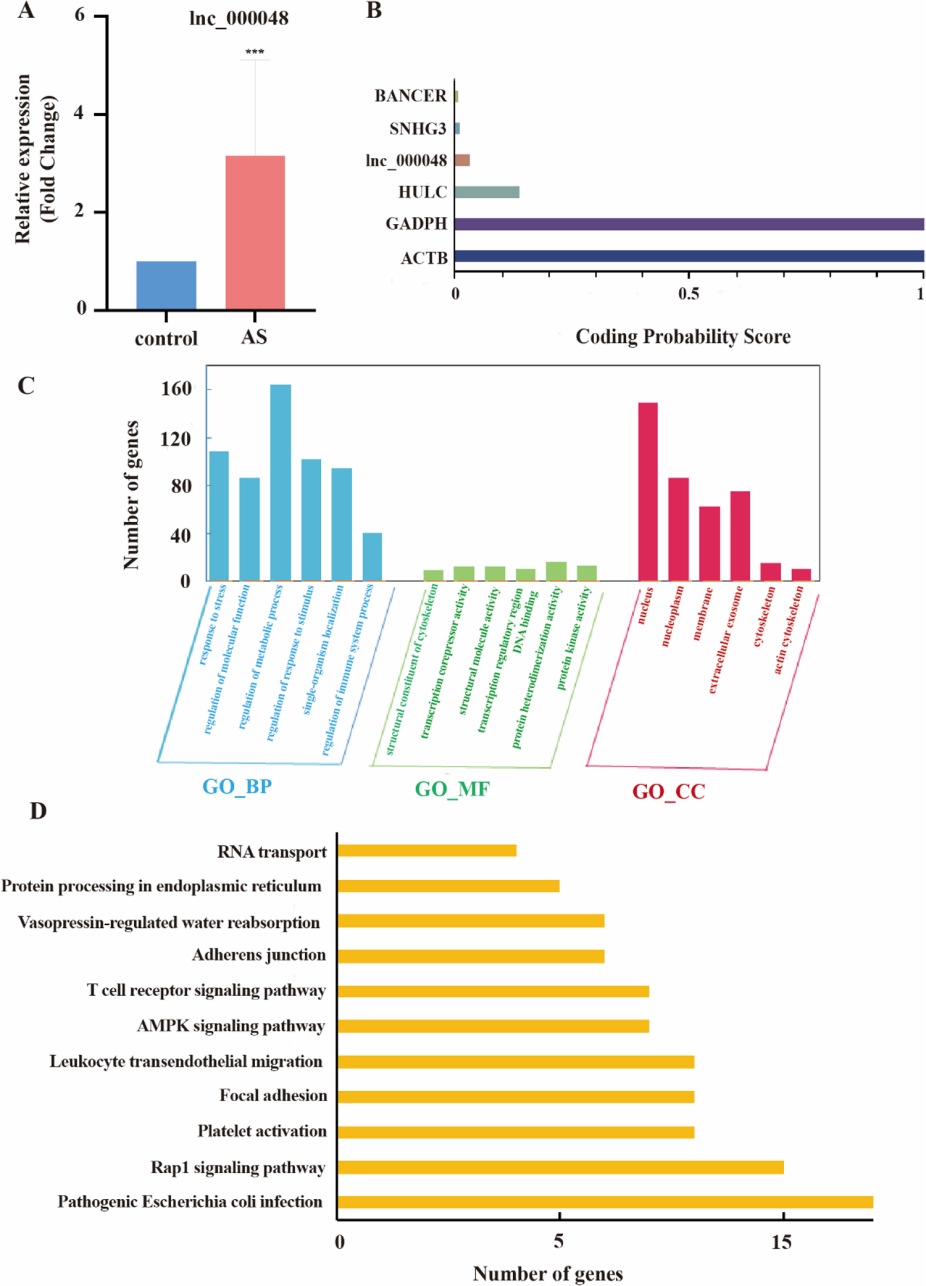
Characteristics of lnc_000048. **A** The expression of lnc_000048 in macrophages and THP-1 macrophage-derived foam cells; **B** CPAT evaluated the ability of coding for lnc_000048, GADPH, ACTB, HULC, SNHG3, and BACER; **C**, **D** GO and KEGG analysis of target genes for lnc_000048

Our previous studies found that up-regulated lnc_000048 may be associated with the rupture of atherosclerotic plaques [18]. qRT-PCR was used to confirm lnc_000048 expression in vitro atherosclerosis model, and the results showed that lnc_000048 was up-regulated in THP-1 macrophage-derived foam cells (Fig. 1A).

We then evaluated the coding potential of lnc_000048 using CPC2, CPAT, and Pfam, and found that lnc_000048 had no coding potential, similar to HOTAIR or NEAT1 (Fig. 1B).

Furthermore, we predicted the target genes of lnc_000048 by using TargetScan, miRanda, and RNAhybrid. The top 10 target genes of lnc_000048 were listed in Supplementary Table S4. Importantly, the biological function of lnc_000048 was assessed using GO and KEGG, and our results indicated that lnc_000048 might be related to immune and platelet aggregation (Fig. 1C, D). Together, these findings imply that the amount of lnc_000048 level is elevated in foam cells produced fromTHP-1 macrophage and probably involved in the process of atherosclerosis.

### Lnc_000048 Increased the Expression Levels of Inflammatory Factors and Matrix Metalloproteinases in THP-1 Macrophage-Derived Foam Cells

We used qRT-PCR and western blotting to analyze the expression of inflammatory cytokines (IL-1, IL-6, and TNF-), as well as matrix metalloproteinases (MMP-2 and MMP-9), in THP-1 macrophage-derived foam cells to assess the phenotypic change. The results showed an increase in inflammatory factors and matrix metalloproteinases in THP-1 macrophage-derived foam cells (Additional file: Fig.S1 A-B). Oil Red O staining indicated the formation of foam cells induced by ox-LDL from THP-1-derived macrophage and the successful establishment of an atherosclerosis model in vitro (Additional file: Fig. S1C).

**Fig. 2:**
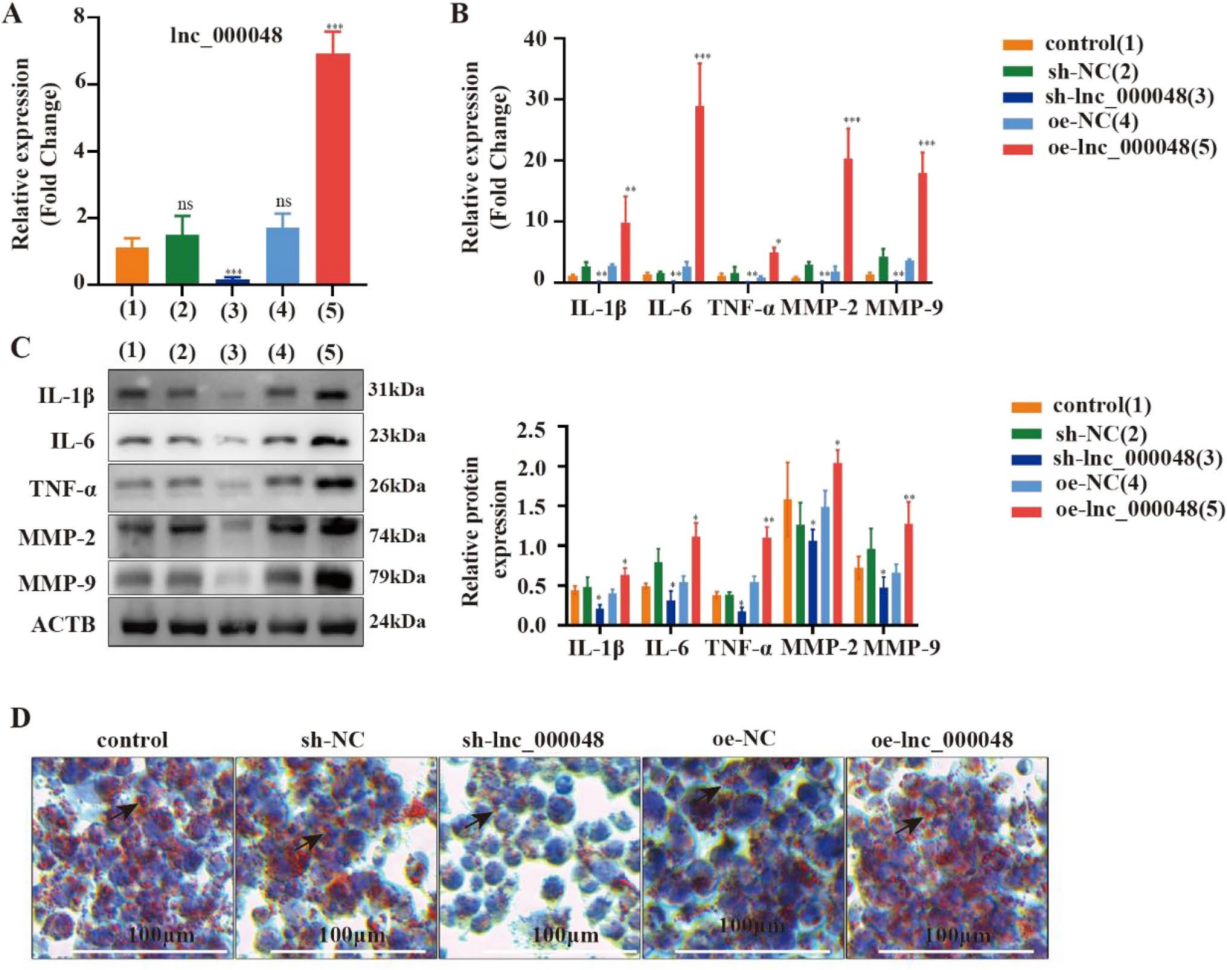
Lnc_000048 accelerated inflammatory responses and collagen degradation matrix in THP-1 macrophage-derived foam cells. **A** qRT-PCR assay was performed in THP-1 macrophage-derived foam cells transfected with lnc_000048 knocked down and overexpressed RNA to evaluate the relative level of lnc_000048. **B** The effects of lnc_000048 on inflammatory cytokine and matrix metalloproteinase gene transcriptional expression were assessed using qRT-PCR among control, sh-lnc_000048, oe-lnc_000048, sh-NC, and oe-NC groups. **C** The effects of lnc_000048 on inflammatory cytokine and matrix metalloproteinase expression were assessed using western blotting among control, sh-lnc_000048, oe-lnc_000048, sh-NC, and oe-NC groups. **D** Effect of lnc_000048 on intracellular lipid accumulation examined using oil red O staining among control, sh-lnc_000048, oe-lnc_000048, sh-NC, and oe-NC groups. *P < 0.05 versus the control group; **P < 0.01. Three times each of the cellular tests were done. The one-way ANOVA method was used to assess comparisons between various groups

To further evaluate the effect of lnc_000048 on atherosclerosis, we constructed and transfected lentiviruses with lnc_000048 knockdown (sh-lnc_000048) or overexpression (oe-lnc_000048). In THP-1 macrophage-derived foam cells, the expression levels of lnc_000048 were different in the sh-lnc_000048 and oe-lnc_000048 groups compared with that in the control group (*p* < 0.05), and there was no difference in the expression level of lnc_000048 between the sh-NC and oe-NC groups (Fig. 2A) (*p* > 0.05).

The effects of lnc_000048 overexpression or knockdown on THP-1 macrophage-derived foam cells were next investigated using western blotting and qRT-PCR. After lnc_000048 knockdown, the expression levels of inflammatory cytokines (IL-1β, IL-6, TNF-α) and matrix metalloproteinases (MMP-2, MMP-9) decreased compared with those in the sh-NC group, following the overexpression of lnc_000048, the release of these factors was higher than that in the oe-NC group (Fig. 2B and C) (*p* < 0.05), highlighting the ability of lnc_000048 to accelerate the inflammation and degradation of collagen in THP-1 macrophage-derived foam cells. The effects of lnc_000048 overexpression and knockdown on intracellular lipid accumulation in THP-1 macrophage-derived foam cells were examined using Oil Red O staining. As shown in Fig. 2D, when compared with the sh-NC group, knockdown of lnc_000048 inhibited intracellular lipid accumulation and overexpression of lnc_000048 increased intracellular lipid accumulation (*p* < 0.05). These findings suggest that lnc_000048 promotes lipid accumulation in foam cells generated from THP-1 macrophages.

Together, all the results suggest that lnc_000048 boosted inflammatory responses s and collagen degradation matrix in THP-1 macrophage-induced foam cells.

### KDM1A Was Essential for ox-LDL-Induced Atherosclerosis in THP-1-Derived Macrophages

**Fig. 3:**
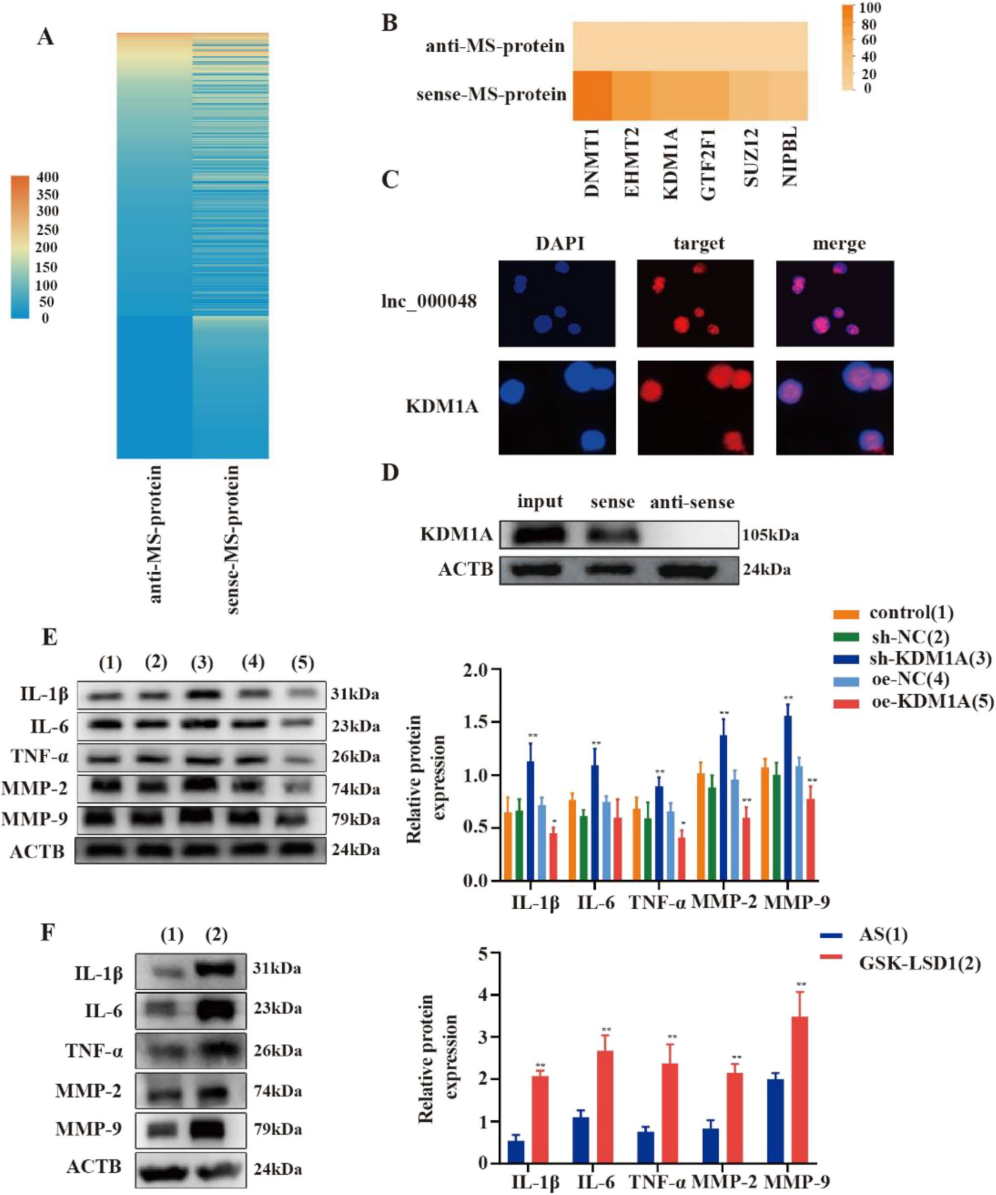
KDM1A was essential for atherosclerosis. **A** Analysis combining mass spectrometry and an RNA pull-down assay to create a heatmap of the proteins bound to lnc_000048. **B** The lnc_000048-binding proteins that are related to epigenetics. **C** Subcellular localization of lnc_000048 and KDM1A in THP-1 cells detected using FISH and immunofluorescence assays. **D** By using an RNA pull-down experiment and western blotting, the interaction between lnc_000048 and KDM1A was confirmed. **E** The effects of KDM1A on inflammatory cytokines and matrix metalloproteinases expression were assessed using western blotting. **F** The effects of an inhibitor of KDM1A (GSK-LSD1) on inflammatory cytokines and matrix metalloproteinases expression were assessed using western blotting. *P < 0.05 versus the control group; **P < 0.01. All cellular experiments were repeated 3 times. Comparison among multiple groups was analyzed using one-way ANOVA

To elucidate the potential mechanism by which lnc_000048 promotes atherosclerosis, we focused on KDM1A, the key player in histone modification. We first identified the potential binding protein of lnc_000048 by RNA pull-down and mass spectrometry. After functional analysis, 319 proteins were identified, and 6 proteins related to epigenetics were obtained (Fig. 3A and B, Additional file: Fig. S2A). Finally, we selected KDM1A, which participates in histone modification and might be involved in the development of atherosclerosis, as an lnc_000048-binding protein. The potential interaction between lnc_000048 and KDM1A was evaluated using the catRAPID bioassay, and the results demonstrated that the interaction between lnc_000048 and KDM1A was likely with a high score of 0.7 (a score of 0.5 is considered as a high possibility of interaction) (Additional file: Fig. S2B).

Furthermore, RNA-FISH assay revealed that lnc_000048 was located in the nuclei of THP-1 cells. Similarly, immunofluorescence results showed that KDM1A was present in the nucleus (Fig. 3C). Spatial localization suggested the possibility of an interaction between lnc_000048 and KDM1A. To further confirm the interaction between lnc_000048 and KDM1A, we conducted WB experiments after RNA pull-down and found that the binding of KDM1A to lnc_000048 was specific (Fig. 3D).

The influences of knockdown or overexpression of KDM1A on THP-1 macrophage-derived foam cells were examined by western blotting. After KDM1A knockdown, the expression levels of inflammatory cytokines (IL-1β, IL-6, and TNF-α) and matrix metalloproteinase (MMP-2 and MP-9) were elevated compared with those in the sh-NC group. Following the overexpression of KDM1A, the release of these factors was lower than that in the oe-NC group (Fig. 3E) (*p* < 0.05), highlighting the ability of KDM1A to reduce the occurrence of inflammation and degradation of collagen in THP-1 macrophage-derived foam cells. More importantly, treating cells with KDM1A inhibitors can significantly accelerate the occurrence of inflammation, suggesting that targeting KDM1A can disturb the process of atherosclerosis (Fig. 3F). Interestingly, we observed no significant change in KDM1A expression level in THP-1 macrophage-derived foam cells, while histone methylation levels, which were regulated by KDM1A, increased. This finding indicated that the activity of KDM1A was altered (Additional file: Fig. S3A–B).

Together, the results shown in Fig. 3A–F and Additional file Fig. S2A-B, S3A–B suggested that in a KDM1A-dependent manner, lnc_000048 involved in the process of atherosclerosis.

### Lnc_000048 Enhanced MAP2K2 Methylation via the Attenuated Activity of KDM1A to Promote Atherosclerosis

**Fig. 4:**
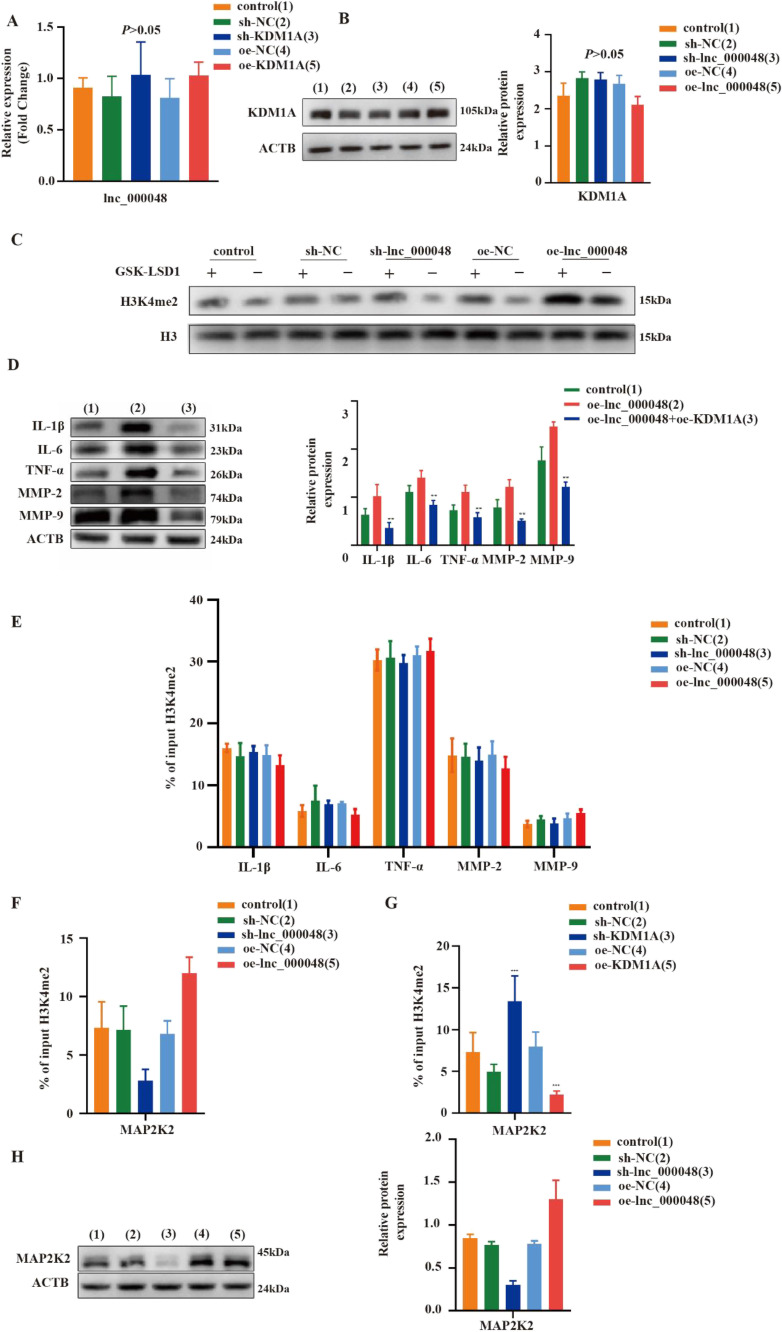
Lnc_000048 elevated the expression of MAP2K2 by attenuating the activity of KDM1A. **A** The effects of KDM1A on lnc_000048 transcriptional expression were assessed using qRT-PCR. **B** The effects of lnc_000048 on KDM1A expression were assessed using western blotting. **C** The effects of lnc_000048 on the activity of KDM1A were assessed using western blotting by combination use of KDM1A inhibitors (GSK-LSD1). **D** The reverse effects of KDM1A on lnc_000048 were assessed using western blotting. **E** Enriched histone 3 lysine 4 dimethylation (H3K4me2) on the promotors of proatherogenic genes. ChIP of H3K4me2 was performed on chromatin from THP-1 macrophage-derived foam cells. H3K4me2 quantification was performed by qRT-PCR analysis of the promotors of IL-1β, IL-6, TNF-α, MMP-2, and MMP-9. **F** The effects of lnc_000048 on enrichment H3K4me2 on the promotors of MAP2K2 were assessed using ChIP and qRT-PCR. **G** The effects of KDM1A on enrichment H3K4me2 on the promotors of MAP2K2 were assessed using ChIP and qRT-PCR. **H** The effects of lnc_000048 on MAP2K2 expression were assessed using WB. **P < 0.01, ***P < 0.001. Three times each of the cellular tests were done. The one-way ANOVA method was used to assess comparisons between various groups

To investigate how lnc_000048 functions via KDM1A to induce atherosclerosis, we first evaluated the effect of the interaction between lnc_000048 and KDM1A on their expression levels. The results showed that the knockdown or overexpression of lnc_000048 did not affect the expression level of KDM1A (Fig. 4A), and the expression level of lnc_000048 was unaffected by KDM1A overexpression or knockdown (Fig. 4B), suggesting that there was no mutual regulation in expression between lnc_000048 and KDM1A.

To further investigate the effect of lnc_000048 on KDM1A, we combined GSK-LSD1 to detect the expression of H3K4me2. The results showed that lnc_000048 attenuated the activity of KDM1A and enhanced the levels of H3K4me2 (Fig. 4C).

Furthermore, to further determine whether the interaction between lnc_000048 and KDM1A contributes to the progression of atherosclerosis, we performed rescue experiments. The results showed that the aggravation of inflammation and collagen degradation caused by lnc_000048 overexpression could be alleviated by KDM1A overexpressed (Fig. 4D). These results further confirmed that KDM1A rescued the pro-atherosclerotic effects induced by lnc_000048.

As a histone demethylase, KDM1A can reduce H3K4me2 levels in the promoter regions of target genes. To further clarify the target molecules of KDM1A, we first performed ChIP to analyze the H3K4me2 levels in the promoter region of inflammatory factors, and the results showed that overexpression or knockdown of lnc_000048 did not affect the levels of histone methylation (Fig. 4E), indicating that lnc_000048 targeted other factors via KDM1A.

In Noonan syndrome, KDM1A could affect the histone methylation level of the MAP2K2 promoter region. By weakening KDM1A, we predicted that lnc_000048 would have an impact on the levels of H3K4me2 in the promoter region of MAP2K2. The results of ChIP showed that H3K4me2 was increased in the promoter region of MAP2K2 in overexpression of lnc_000048 (Fig. 4F). To further clarify whether lnc_000048 affects MAP2K2 through KDM1A, we analyzed the levels of histone methylation of MAP2K2 by knocking down or overexpressing KDM1A. The results demonstrated that knockdown KDM1A and overexpressed lnc_000048 had similar effects (Fig. 4G). Western blotting showed the similar results (Fig. 4H, Additional file: Fig. S4B).

Together, the results from multiple approaches shown in Fig. 4A–H and Additional file Fig. S4B suggested that lnc_000048 could enhance MAP2K2 function by attenuating the histone demethylase activity of KDM1A to promote atherosclerosis.

### Lnc_000048 Indirectly Enhanced the Phosphorylation of ERK

Atherosclerosis develops in large part as a result of the MAPK pathway, and our research found that the expression level of MAP2K2 was elevated during atherosclerosis development (Additional file: Fig. S4A). As an important member of the MAPK pathway, MAP2K2 activates ERK, leading to its phosphorylation. We further measured the levels of total and phosphorylated ERK (p-ERK) and found that the expression level of total ERK was unchanged and p-ERK was enhanced in THP-1 macrophage-derived foam cells (Additional file: Fig. S4A).

**Fig. 5:**
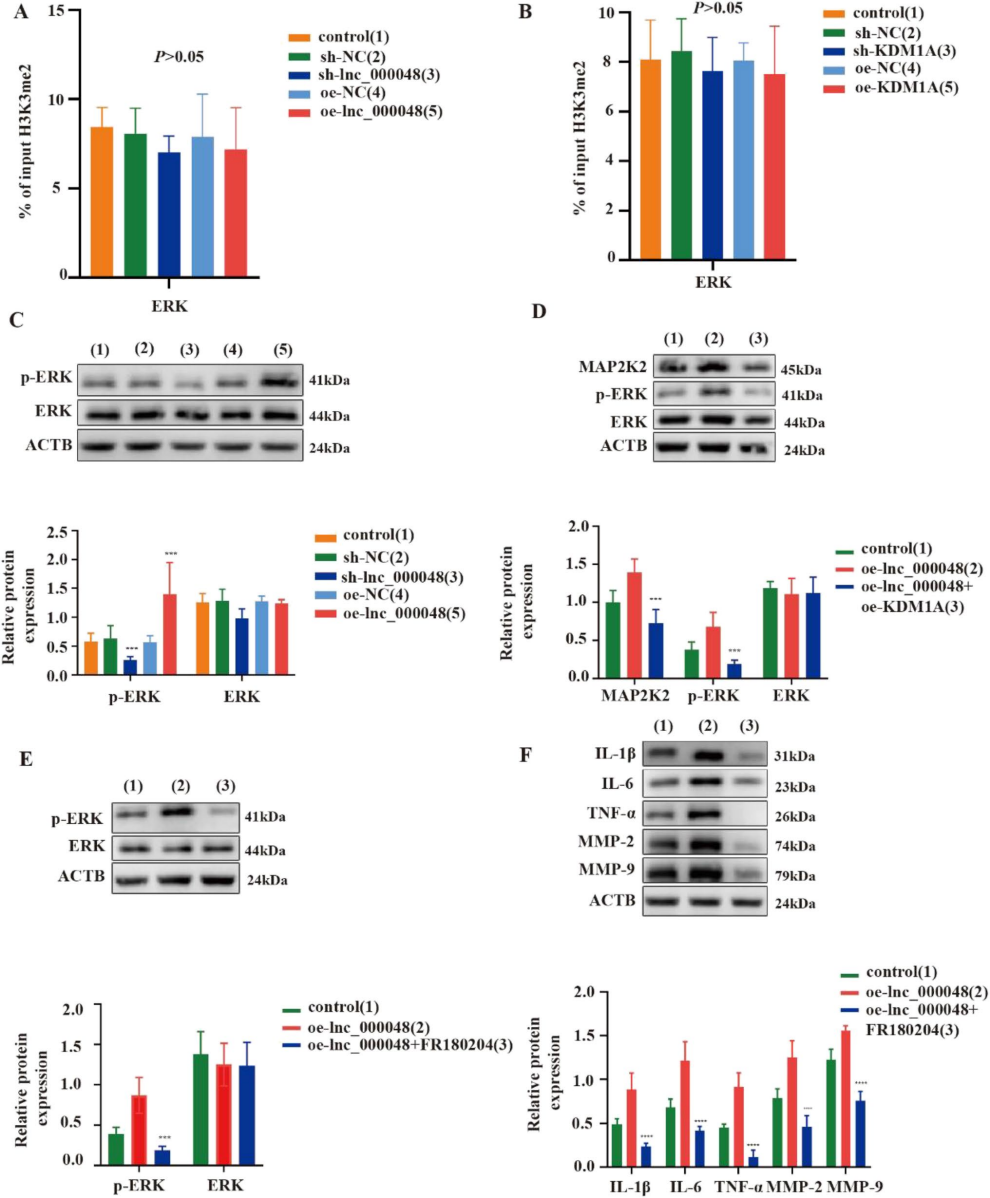
Lnc_000048 enhanced the phosphorylation of ERK. **A** The effects of lnc_000048 on the enrichment of H3K4me2 on ERK promotors were assessed using ChIP and qRT-PCR. **B** The effects of KDM1A on the enrichment of H3K4me2 on ERK promotors were assessed using ChIP and qRT-PCR. **C** The effects of lnc_000048 on ERK and p-EKR expression were assessed using western blotting. **D** The reverse effects of KDM1A on changes in MAPKs pathway induced by lnc_000048 were assessed using western blotting. **E**, **F** The reverse effects of MAPKs pathway inhibitor (FR180204) on lnc_000048 were assessed using western blotting: ERK and p-ERK(**E**) and inflammatory cytokines and matrix metalloproteinases (**F**). ***P < 0.001; ****P < 0.0001. Three times each of the cellular tests were done. The one-way ANOVA method was used to assess comparisons between various groups

Our previous study found that lnc_00048 increased the levels of histone methylation in the promoter region of MAP2K2. We performed a ChIP experiment to explore whether lnc_000048 plays a similar role in ERK. The results showed that there were no significant changes of H3K4me2 in ERK among knockdown or overexpressed lnc_000048 and KDM1A groups (Fig. 5A and B), which suggested that phosphorylation of ERK was induced by lnc_000048 in an indirect manner. Western blotting confirmed that overexpression of lnc_000048 promoted the expression of MAP2K2 and p-ERK, whereas knockdown of lnc_000048 had the opposite effect. However, lnc_000048 overexpression or knockdown had little effect on the overall amount of ERK expression in THP-1 macrophage-derived foam cells (Fig. 4H and Fig. 5C), suggesting that lnc_000048 interferes with the phosphorylation of ERK by affecting MAP2K2 rather than total ERK. In addition, we found that MAP2K2 expression and ERK phosphorylation levels increased in the KDM1A knockdown group (Additional file: Fig. S4B). More importantly, the increased MAP2K2 and p-ERK levels caused by lnc_000048 overexpression could be alleviated by KDM1A overexpression (Fig. 5D). These results further confirmed that KDM1A rescued the pro-atherosclerotic effects induced by lnc_000048.

**Fig. 6:**
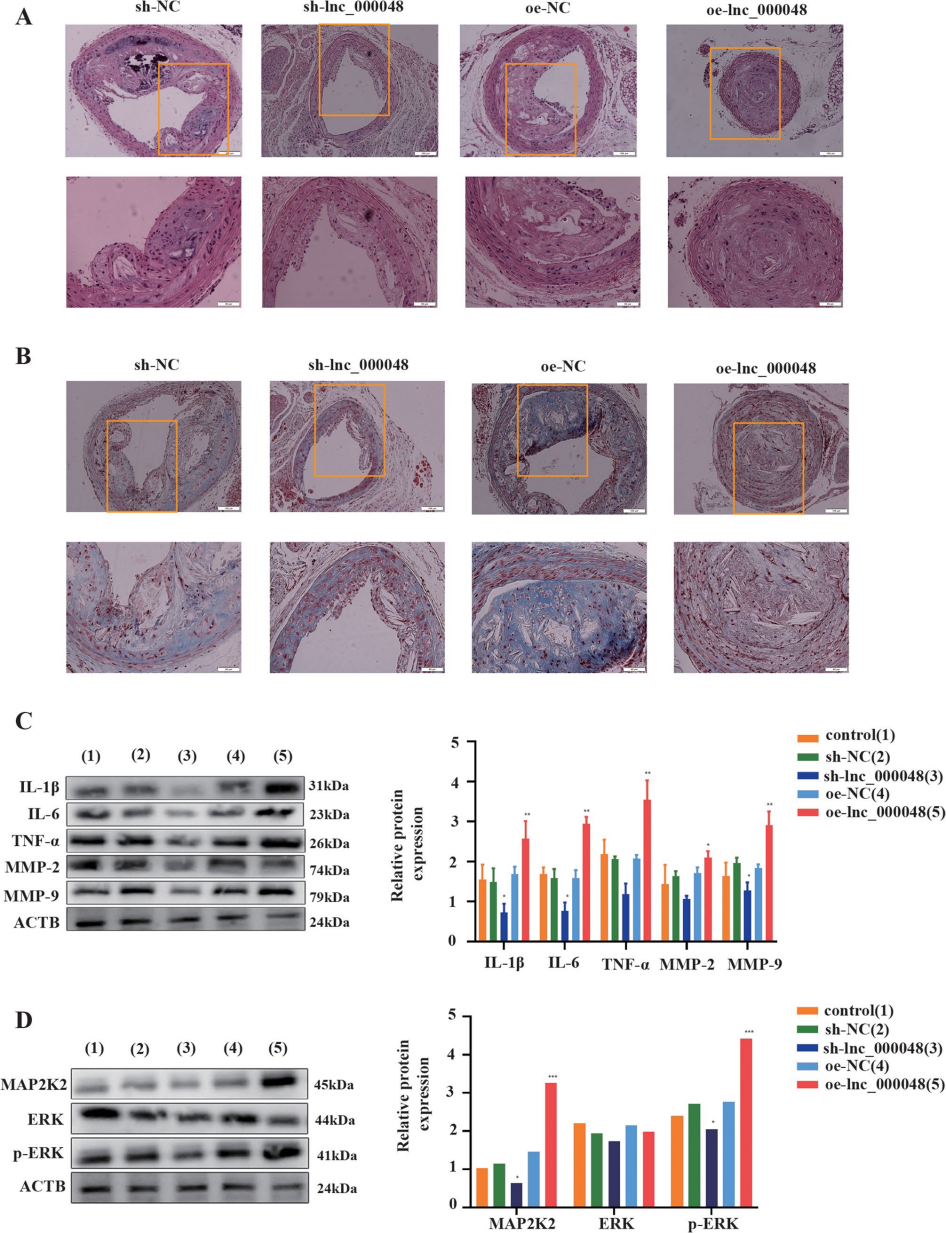
Lnc_000048 promoted atherosclerosis progression in ApoE–/– mice. **A** Analysis of carotid plaque development in ApoE-/- mice with atherosclerosis following treatment with sh-lnc_000048 or oe-lnc_000048 using HE staining. **B** Masson staining analysis of collagen degradation of carotid plaque in ApoE-/- mice after sh-lnc_000048 or oe-lnc_000048 treatment. **C** The effects of lnc_000048 on inflammatory cytokine and matrix metalloprotein expression were assessed using western blotting. **D** The effects of lnc_000048 on the expression of factors of MAPKs pathway assessed using western blotting. *P < 0.05; **P < 0.01; ***P < 0.001. All experiments were repeated three times. The one-way ANOVA method was used to assess comparisons between various groups

Furthermore, we used the MAPK inhibitor, FR180021, to observe the role of the MAPK pathway in atherosclerosis. The results demonstrate that FR180712 significantly inhibited lnc_000048-induced activation of the MAPK signaling pathway and the release of inflammatory factors and matrix metalloproteins in THP-1 macrophage-derived foam cells (Fig. 5E and F).

Together, the results shown in Fig. 5A–F and Additional file Figure S4A-B indicated that lnc_000048 participated in atherosclerosis through the MAPK pathway.

### Lnc_000048 Accelerated the Inflammation and Degradation of Collagen in ApoE-/- Mice with Carotid Atherosclerosis

To confirm the role of lnc_000048 in an in vivo mouse model, we successfully simulated carotid atherosclerosis through partial carotid ligation with or without lentivirus vector infection for the knockdown or overexpression of lnc_000048 in ApoE-/- mice. HE staining showed that the structure of the carotid intima was disordered, inflammatory cells were aggregated, and plaques were formed in the carotid artery, at the same time, we have tested the level of KDM1A in the plaque and the results showed that the level of KDM1A has no changes, which is consistent with our results in vitro (Additional file: Fig. S5A and Fig. S5B). The qRT-PCR results demonstrated that knockdown and overexpression of lnc_000048 were effective in the release of inflammatory factors and matrix metalloproteinases in atherosclerotic plaques (Additional file: Fig. S5C).

The results of HE staining of the carotid artery showed that ApoE-/- mice with lnc_000048 knockdown exhibited less plaque; however, in the overexpressed lnc_000048 group, the structure of the carotid intima was disturbed, the intima thickened, and the plaque volume increased significantly (Fig. 6A). Masson staining indicated that the volume of carotid plaque increased significantly and decreased, and collagen degradation was more serious in ApoE-/- mice overexpressing lnc_000048. However, lnc_000048 knockdown exhibited the opposite effect, suggesting that lnc_000048 can accelerate the process of atherosclerosis (Fig. 6B).

Finally, the overexpression of lnc_000048 enhanced the expression of inflammatory factors and matrix metalloproteinases in carotid plaque samples, activating the MAP2K2-ERK axis, according to the results of our western blotting (Fig. 6C and D).

Together, the results from our studies in ApoE-/- mice validated that lnc_000048 promoted atherosclerosis by activating the lnc_000048/MAP2K2/ERK axis.

## Discussion

**Fig. 7:**
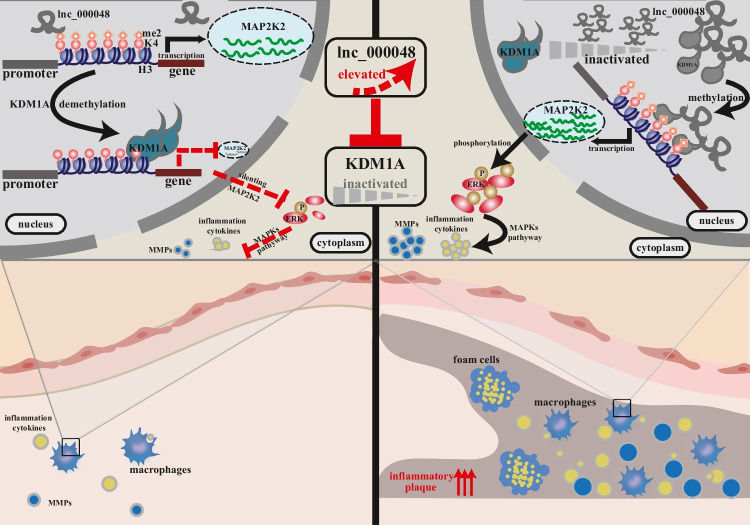
Schematic depiction of lnc_000048 /KDM1A/MAP2K2/ERK pathway in atherosclerosis and plaque

LncRNAs participate in various stages of atherosclerosis by mediating histone modification [32–34]. In this study, we presented a new perspective that lnc_000048 participated in atherosclerosis via epigenetic modifications caused by its interaction with KDM1A. Our results suggested that lnc_000048 weakened the enzymatic activity of KDM1A to increase histone methylation levels in the promoter region of MAP2K2 and stimulate the phosphorylation of ERK, resulting in increased expression levels of inflammatory cytokines and matrix metalloproteinases (Fig. 7). These findings suggested that lnc_000048 /KDM1A/MAP2K2/ERK was a key regulatory axis in atherosclerosis progression.

Our study found that lnc_000048 levels were increased in ox-LDL-treated macrophages, which was consistent with our previous RNA-seq findings. Lnc_000048 is a long non-coding RNA with a transcript of 1.128 kb, functional enrichment suggested that lnc_000048 may be involved in atherosclerotic biological processes, such as inflammation, platelet-activation, and epigenetic modification. However, the specific role of lnc_000048 in atherosclerosis remains unknown.

LncRNAs can participate in histone modification and regulate gene expression by recruiting proteins that regulate disease progression [35]. However, current studies on histone modification by lncRNAs are mostly focused on cancer, and their role in atherosclerosis is still in its infancy. In our study, through RNA pull-down and mass spectrometry analyses, we found that lnc_000048 could bind KDM1A to participate in the development of atherosclerosis through histone modification.

Subsequent subcellular localization showed that both lnc_000048 and KDM1A localize to the nucleus, suggesting the possibility of a spatial interaction. Subcellular localization of lncRNAs can indicate their possible mode of action in cells such as competing endogenous RNA (ceRNA) [36]. LncRNAs can induce epigenetic modifications and regulate the transcription of target genes by co-locating with proteins in the nucleus [37]. Ren et al. proposed that lncRNA PLACT1 and hnRNPA1 co-localized in the nucleus and then induced an increase of H3K27, which reduces the transcription level of IκBα [38]. We further confirmed the physical binding relationship between lnc_000048 and KDM1A using the RNA pull-down combined WB technique.

Furthermore, we found that lnc_000048 recruited KDM1A and inhibited its demethylase activity of KDM1A, which further mediated the enrichment of H3K4me2 in the promoter region and activated the expression of MAP2K2. LncRNAs can recruit histone modification enzymes as well as transcription factors to specific genomic sites and exert transcriptional inhibition or activation of downstream target genes [39, 40]. For example, lncRNA-Zeb1-AS1 activates ZEB1(a transcription factor) through epigenetic activation, indirectly regulates downstream target molecules of ZEB1, and is eventually involved in tumors [41]. Pandey et al. found that lncRNA Kcnq1ot could specifically interact with H3K9- and H3K27-specific histone methyltransferase G9a and PRC2 complexes, ultimately promoting increased levels of H3K27me3 and H3K9me3 [42]. In our study, we found that lnc_000048 could recruit and affect the demethylase activity of KDM1A, eventually regulating the expression of downstream target genes. These results were similar to those reported by Choi et al. [14]. Future studies will be of interest to explore the bonding point between lnc_000048 and KDM1A to further investigate the mechanism of lnc_000048 in the process of atherosclerotic plaques.

As an important histone demethylase, KDM1A regulates gene transcription in an epigenetic manner by altering histone or non-histone methylation levels in the promoter region [43–45]. Our study found that lnc_000048 participated in MAP2K2 transcription by affecting histone methylation in the promoter region of MAP2K2, which was caused by KDM1A. LncRNAs can recruit KDM1A to participate in the biological processes of tumors and angiogenesis. Pu et al. found that MAGI2-AS3 might attract KDM1A to encourage the demethylation of H3K4me2 in the RACGAP1 promoter, which would ultimately lower the level of RACGAP1 expression and control the proliferative, migratory, and invading capacities of HCC cells [46]. Di Zhao et al. reported that lncRNA-HOTAIR interacted with KDM1A to induce partial transcriptomic reprogramming in the endothelial cell [47]. Our results are consistent with Kent’s report that silencing KDM1A leads to an increased expression level of MAP2K2, which activates downstream MAPK pathway factors, such as the phosphorylation of ERK [48]. However, these therapeutic targets still need more validation in vivo trials in future.

Finally, the results showed that lnc_000048 accelerated the formation and instability of atherosclerotic plaques in ApoE-/- mice in vivo. Overexpression of lnc_000048 enhances the accumulation of inflammatory cells and collagen degradation in plaques.

## Conclusions

In conclusion, our study found that lnc_000048 significantly induced the release of matrix metalloproteinases and inflammatory factors in models of atherosclerosis. Mechanistically, lnc_000048 affected histone demethylase activity by recruiting KDM1A, promoting the transcription of MAP2K2 by accumulating H3K4me2 in the promoter region, further accelerating the phosphorylation of ERK, and eventually promoting downstream inflammatory factors. More importantly, our results suggested that lnc_000048 affected atherosclerosis progression by inhibiting KDM1A activity rather than its expression. Our results indicated how lnc_0000048 promoted atherosclerosis and provide new potential strategies to target the lnc_000048 /KDM1A/MAP2K2/ERK axis to inhibit the progression of atherosclerosis.

## Supplementary Information

Below is the link to the electronic supplementary material.

Supplementary file1 (DOCX 1.97 MB)

## Data Availability

The data that support the findings of this study are available from the corresponding author upon reasonable request.

## Abbreviations

lncRNAs: Long non-coding RNAs
qRT-PCR: Quantitative Real-time PCR
ChIP: Chromatin immunoprecipitation
MS: Mass spectrometry
FISH: Fluorescence in situ hybridization
H3K4me2: Histone 3 lysine 4 dimethylation
