# Supplementary material for: Lnc_000048 Promotes Histone H3K4 Methylation of MAP2K2 to Reduce Plaque Stability by Recruiting KDM1A in Carotid Atherosclerosis
Source: Mol Neurobiol. 2023 Jan 23;60(5):2572–86. doi: 10.1007/s12035-023-03214-0 (PMC10039837; doi:10.1007/s12035-023-03214-0)
Supplement: Supplementary file 1 — Supplementary file1 (DOCX 1.97 MB) [file 12035_2023_3214_MOESM1_ESM.docx]

**SUPPLEMENTARY MATERIAL**

**Supplementary Methods**

***Bioinformatics Analysis***

Lnc_000048 was identified from the RNA-seq and gene expression data which can be accessed by the NCBIs Gene Expression Omnibus (GEO, <http://www.ncbi.nlm.nih.gov/geo/>). The GEO Series accession number is GSE173719. Targetscan, miRanda, RNAhybrid, DAVID database and KOBAS database were used to infer the potential functions of the targets of lnc_000048 in terms of biological process (BP), cellular component (CC), and molecular function (MF) GO annotations and KEGG pathways. We next analyzed the protein-coding potential of lnc_000048 by using Coding Potential Calculator (http://cpc.cbi.pku.edu.cn/) and Coding Potential Assessment Tool (http://lilab.research.bcm.edu/cpat/index.php) based on sequence features. Conserved Domain Database (https://www.ncbi.nlm.nih.gov/cdd/) and Pfam (http://pfam.xfam.org/) were used to analyze the conserved domains. CatRAPID (<http://s.tartaglialab.com/page/catrapid_group>) was used to evaluate the binding potential of lnc_000048 to KDM1A. R package tools were used to visualize the results.

***Cell culture and Drug treatment***

The human mononuclear cell line (THP-1) was purchased from Procell Life Science&Technology Co.,Ltd (Wuhan, China). The cells were cultured in a humidified 5% CO_2_ atmosphere at 37°C in RPMI 1640 (Procell Life Science&Technology Co.,Ltd, Wuhan, China) with 10% FBS (GIBCO, Carlsbad Co.,Ltd, CA, USA) and 0.05Mm β-mercaptoethanol (Procell Life Science&Technology Co.,Ltd, Wuhan, China) as well as 1% penicillin-streptomycin solution (Procell Life Science&Technology Co.,Ltd, Wuhan, China). Cell lines were systematically tested and were negative for mycoplasmas. THP-1 cells were cultured in 6-well plates or 10cm^2^ dishes as required to exponential phase. After that, the cells were treated with 100 nmol/L PMA (Sigma-Aldrich Chemical Company, St. Louis, MO, USA) for 48h to be induced from monocytes into macrophages. Then 100 μg/mL ox-LDL (Yiyuan biotechnology, Guangzhou, China) was added into serum-free RPMI 1640 medium for 48 h to construct the atherosclerosis model in vitro for subsequent experiments. According to the requirements of the experiment, the model cells were pretreated with signaling pathways inhibitors or enzyme inhibitors, including MEK/ERK inhibitors (FR 180204, terminal concentration 1 µM) and GSK-LSD1 (final concentration 5 µM). All inhibitors were purchased from MCE.

***Lentivirus and plasmid transfection***

The vectors that used to construct the lentiviral vector was GV493 (lentivirus gene silencing vector of shRNA for lnc_000048), GV367 (lentivirus gene overexpression vector for lnc_000048), GV102 (lentivirus gene silencing vector of shRNA for KDM1A) and GV657 (lentivirus gene overexpression vector for KDM1A). The lentiviral vectors were constructed by Genechem Co.,Ltd. (Shanghai, China). Lentiviral packaging were performed by co-transfection of 293T cells with recombinant lentivirus vectors, pHelper 1.0 vectors and pHelper 2.0 vectors. THP-1 cells were classified into the following groups according to different transfections: sh-NC group (transfected with irrelevant shRNA sequence lentiviral vector); sh-lnc_000048 group (transfected with lnc_000048 shRNA lentiviral vector); oe-lnc_000048 group (transfected with lnc_000048 overexpression lentiviral vector); sh-KDM1A (transfected with KDM1A shRNA lentiviral vector); oe-KDM1A (transfected with KDM1A overexpression lentiviral vector); oe-lnc_000048 + oe-KDM1A (co-transfected with lnc_000048 overexpression and KDM1A overexpression lentiviral vector). The THP-1 in the logarithmic growth phase were made into a cell suspension of 5×10^5^ cells/ml. Next, the cell suspension was then seeded in a 12-well plate with 1 mL per well and incubated overnight at 37℃. The virus was added to the cells for infection according to the manufacturer’s instructions. These cells were treated with puromycin (2μg/mL) for 48h to obtain stably transfected cell lines for subsequent experimentation. For plasmid transfection, the Lipofectamine 2000 (Invitrogen/Thermo Fisher Scientific, Waltham, MA, USA) transfection protocol was adapted as follows: 250ng of DNA + 0.5μl of Lipofectamine 2000, for a 12-well multiwell format.

***RNA isolation and quantification***

In short, total RNAs were extracted directly from cells using TRIzol (Invitrogen, Grand Island, NY, USA). 1 µg total RNA was subjected to reverse transcription using a Goldenstar™ RT6 cDNA Synthesis Kit (TSK302, TSINGKE). Quantitative real-time PCR (qRT-PCR) was conducted using a 2×T5 Fast qPCR Mix (TSE301, TSINGKE) to detect the expression levels of lncRNAs and mRNA. The expression levels were normalized to ACTB expression level. Primers used for the genes of interest are listed in Supplementary Table S1.

***Protein extraction and western blotting analysis***

Cells were lysed in RIPA buffer (Thermo Scientific) supplemented with PMSF (Solarbio, USA). A total of 25µg of the whole protein extract was separated on a 4–12% Tris Protein Gel (Sangon Biotech, Shanghai) and transferred using a Bio-Rad Blotting System (BIO-RAD, USA). After blocking, membranes were immunoblotted with anti-ACTB, anti-IL-1β, anti-IL-6, anti-TNF-α, anti-MMP-2, anti-MMP-9, anti-MAP2K2, anti-KDM1A, anti-ERK, anti-p-ERK, anti-H3, and anti-H3K4me2 (Abcam, USA) overnight at 4°C, then washed and incubated with a corresponding HRP-conjugated secondary antibody for 1 h at room temperature and visualized using a chemiluminescence system (UVITEC, UK). Quantifications of protein amounts was performed relative to ACTB using ImageJ software.

***RNA pull-down assay and mass spectrometry analysis***

Biotin-labelled lnc_000048 (sense) and control (antisense) probes (Supplementary Table S2) were synthesized by KAXU (Shanghai, China). RNA pull-down assays were performed as previously described. Briefly, ~10^7^ cells were washed in ice-cold phosphate-buffered saline, lysed in 500 μL cell lysis buffer, and incubated with 3 μg biotinylated DNA oligo probes, at room temperature for 3 h. A total of 150 μL Pierce Nucleic-Acid Compatible Streptavidin Magnetic Beads washed by Wash Buffer were and further incubated at room temperature for 1 h. The beads were briefly washed five times with Wash Buffer. 50 μL Biotin Elution buffer was used for protein elution. The bound proteins in the pull-down materials were analyzed using mass spectrometry or western blotting.

5x SDS PAGE loading buffer was added to the sample, and then the sample was boiled at 100 ° C for 10min and then quickly transferred to ice for 2 minutes. SDS-PAGE was used for electrophoresis. Run electrophoresis with 20μL protein solution, stop electrophoresis at the place where the concentrated gel ran out and just entered the separation gel 1cm, cut the gel and add 1ml ddH2O. Finally, the strip was cut off for mass spectrometry analysis.

***Oil red O staining***

To assess the accumulation of lipid in cells, the Oil Red O Stain Kit was performed as manufacturer's instructions (G1262; Solarbio; Beijing, China). In short, In short, THP-1 cells were cultured in 6-well plates using sterilized coverslips. After treatment, cells were fixed in ORO Fixativ for 25 min and then wash them twice with distilled water. Add 60% isopropanol and soak for 30s，then stained with Oil Red O staining solution for 10 min, and stained with Mayer hematoxylin stainingsolution and counterstain the nucleus for 1 min. Intracellular lipids were viewed as red and nuclei were viewed as blue when observed under a microscope; photographs were collected and analyzed using an image analysis system.

***Fluorescence in situ hybridization (FISH)***

Fluorescence in situ hybridization assays were performed as manufacturer's instructions (C10910; RiboBio; Guangzhou).FISH was used to assess lnc_000048 distribution in THP-1 cells. Briefly, the cells were allowed to adhere to coverslips, which were then incubated in a 24-well plate (5 × 10^3^ cells/well) for 24 h. Next, the cells were fixe in 4% paraformaldehyde and phosphate buffer saline containing 0.5% Triton X-100 was added. The pre-hybridization solution, lnc_000048 probe, and hybridization solution were then added to the cells. The hybridization area on the coverslip was stained with 4', 6-diamidino-2-phenylindole (DAPI) and observed under a laser scanning confocal microscope.

***Immunofluorescence***

Immunofluorescence was used to assess the distribution of KDM1A in THP-1 cells. The cells were fixed in 4% paraformaldehyde, added to phosphate buffered saline containing 0.5% Triton X-100, and then incubated with anti-KDM1A at 4℃ overnight. Alexa Fluor 647 labeled IgG was used as the secondary antibody. DAPI was used to visualize cell nuclei, and fluorescent images were obtained using a fluorescence microscope.

***HE staining***

HE staining was performed as described previously.The specimens were gently sliced into 5-mm sections. The obtained sections were dewaxed routinely with xylene, stained with hematoxylin for 5-10 min, stained with eosin dye for 1 min, and observed under an ordinary optical microscope. Images were captured using ImageJ image processing software.

***Masson staining***

Masson staining was conducted using a ready–to-use kit (Masson's Trichrome Stain Kit, Solarbio). Briefly, tissue sections (thickness, 5 μm) were cut. After deparaffinization and rehydration, the slides were immersed in Weigert’s Iron Hematoxylin solution for 5 min. The slides were then differentiated with Acid Alcohol Differentiation Solution for 2s and washed with tap water for 20 min. Next, the slides were stained with Ponceau-Acid Solution for 5 min and differentiated in Phosphomolybic Acid Solution for 1min. Then Aniline Blue Solution for 2min, rinsed in distilled water, incubated in Acetic Acid Working Solution for 1 min. Finally, the slides were dehydrated, transparentized, and mounted. Images were captured using ImageJ image processing software.

***Statistical analysis***

Data represent the mean ± standard error (SD). Comparisons between the controls and treatment groups were performed using one-way ANOVA. The Mann–Whitney U test or Student’s t-test was used to compare continuous variables between patients and controls. Statistical significance was defined as *P* < 0.05 for all tests. Statistical analyses were performed using Statistical Package for the Social Sciences software version 17.0 (SPSS Inc., Chicago, IL, USA) and GraphPad Prism 6.

**Supplementary Tables**

**Table S1 Primer sequences for qRT-PCR**

| Genes | 5’-3’ | **Primer sequences** |
| --- | --- | --- |
| Lnc_000048 | Forward | TGGGCGGGATTCTGACTTAGAGG |
|  | Reverse | GGTGTATGTGCTTGGCTGAGGAG |
| KDM1A | Forward | GACTTCTTGGCAGAGTTGTC |
|  | Reverse | GTGAAAGAGTTGCAGATCC |
| IL-1β | Forward | CGGGATCCGCACCTGTACGATCACTGAAC |
|  | Reverse | CCGCTCGAGTGGGTACAGCTCTCTTTAGG |
| IL-6 | Forward | AGGGAGAGCCAGAACACAGA |
|  | Reverse | GAGTTTCCTCTGACTCCATCG |
| TNF-α | Forward | GTGCTTGTTCCTCAGCCTCT |
|  | Reverse | ATCACTCCAAAGTGCAGCAG |
| MMP-2 | Forward | CTCATCGCAGATGCCTGGAA |
|  | Reverse | TTCAGGTAATAGGCACCCTTGAAGA |
| MMP-9 | Forward | ACGCACGACGTCTTCCAGTA |
|  | Reverse | CCACCTGGTTCAACTCACTCC |
| ACTB | Forward | GCGGACTATGACTTAGTTGCGTTACA |
|  | Reverse | TGCTGTCACCTTCACCGTTCCA |

**Table S2 Lnc_000048 primer sequences for RNA pull-down**

| Genes | 5’-3’ | **Primer sequences** |
| --- | --- | --- |
| sense | Forward | TAATACGACTCACTATAGGGACCCAGAAGCAGGTCGTCT |
|  | Reverse | CACCTGCCGAATCAACTAGCC |
| antisense | Forward | ACCCAGAAGCAGGTCGTCT |
|  | Reverse | TAATACGACTCACTATAGGGCACCTGCCGAATCAACTAGCC |

**Table S3 Primer sequences for ChIP**

| Genes | 5’-3’ | **Primer sequences** |
| --- | --- | --- |
| MAP2K2 | Forward | GAGGAGACGGAGGGATGAAG |
|  | Reverse | GGGGTCTTACGGGTTCTCTC |
| ERK | Forward | TGTACTTGGTGACGGCCTTA |
|  | Reverse | CATTACAACAAGCGCTCGAC |
| IL-1β | Forward | ATGCACTGGGAGACAATTCC |
|  | Reverse | CTCCCTCCACCTTCTTCCTC |
| IL-6 | Forward | TCGTGCATGACTTCAGCTTT |
|  | Reverse | GCGCTAAGAAGCAGAACCAC |
| TNF-α | Forward | CAGGCAGGTTCTCTTCCTCT |
|  | Reverse | GCTTTCAGTGCTCATGGTGT |
| MMP-2 | Forward | CTGCATCCAGACTTCCTCAG |
|  | Reverse | GTCCTGGCAATCCCTTTGTA |
| MMP-9 | Forward | CTCTGCCAGCTGCCTGTC |
|  | Reverse | CTCTGCCAGCTGCCTGTC |
| MYO | Forward | AGCATGGTGCCACTGTGCT |
|  | Reverse | GGCTTAATCTCTGCCTCATGAT |

**Table S4 The top 10 target genes of lnc_000048**

| lncRNA_ID | mRNA | *r* | *P* value |
| --- | --- | --- | --- |
| Lnc_000048 | GUK1 | 0.999103916 | <0.00001 |
| Lnc_000048 | MORF4L1 | 0.999034955 | <0.00001 |
| Lnc_000048 | CPT1A | 0.998870515 | <0.00001 |
| Lnc_000048 | LRIF1 | 0.998073475 | <0.00001 |
| Lnc_000048 | ZNF185 | 0.997796952 | <0.00001 |
| Lnc_000048 | CALD1 | 0.997687487 | <0.00001 |
| Lnc_000048 | TRIQK | 0.997686408 | <0.00001 |
| Lnc_000048 | OSBP2 | 0.997686319 | <0.00001 |
| Lnc_000048 | RHEB | 0.997656002 | <0.00001 |
| Lnc_000048 | ARPC4-TTLL3 | 0.99756621 | <0.00001 |

**Supplementary Figures**

**
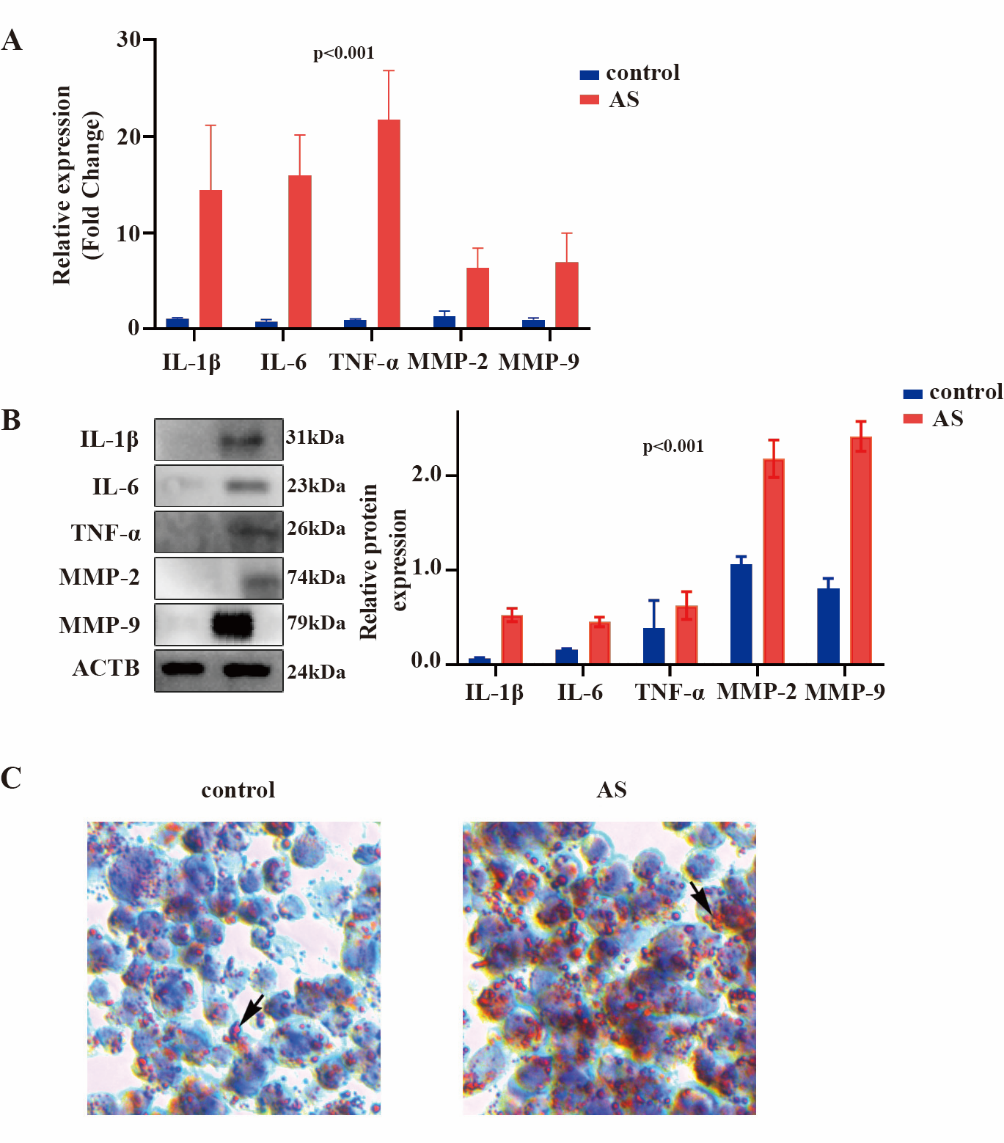
**

**Figure S1. Changes of phenotypic in atherosclerosis**. (A) qRT-PCR assay was performed in THP-1 macrophage-derived foam cells to evaluate the relative levels of inflammatory cytokine and matrix metalloproteinase gene transcriptional expression in AS. (B) The relative levels of inflammatory cytokine and matrix metalloproteinase in AS were assessed using western blotting. (C) Intracellular lipid accumulation examined using oil red O staining. All cellular experiments were repeated 3 times. Comparison among multiple groups was analyzed using Mann-Whitney test.

**
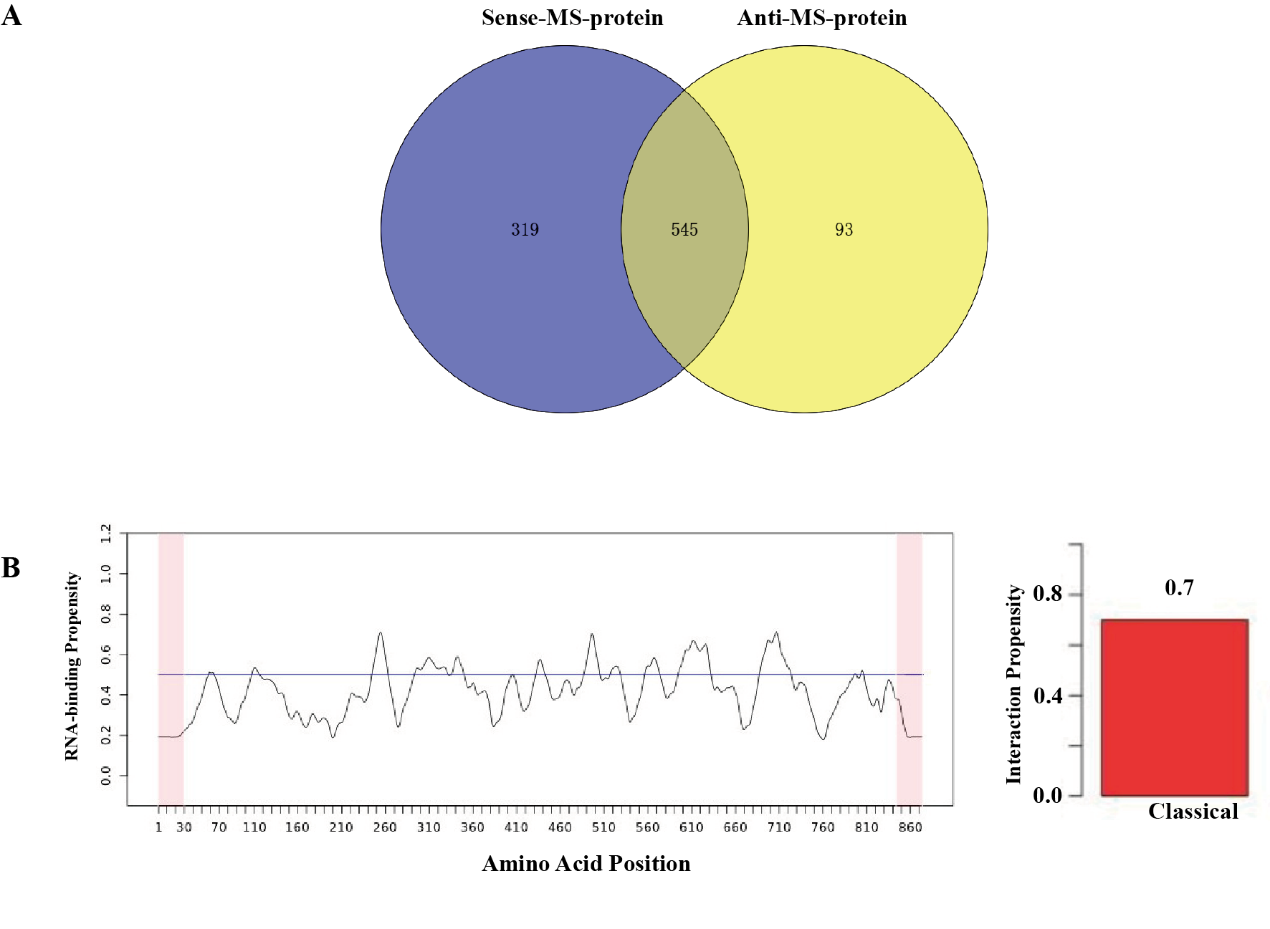
**

**Figure S2. The interaction of lnc_000048 to KDM1A.** (A) Veen map of the proteins bound to lnc_000048 using RNA pull-down assay and mass spectrometry analysis. (B) The propensity of interaction between lnc_000048 to KDM1A was evaluated by catRAPID.

**
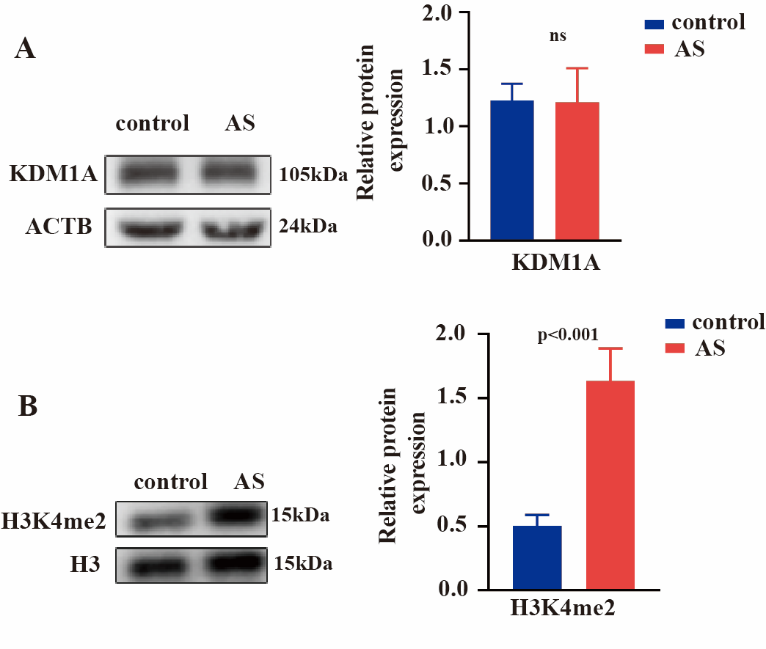
**

**Figure S3. Changes of KDM1A and its role in atherosclerosis**. (A) The relative level of KDM1A in AS were assessed using western blotting. (B) The relative level of H3K4me2 in AS were assessed using western blotting. All cellular experiments were repeated 3 times. Comparison among multiple groups was analyzed using Mann-Whitney test.

**
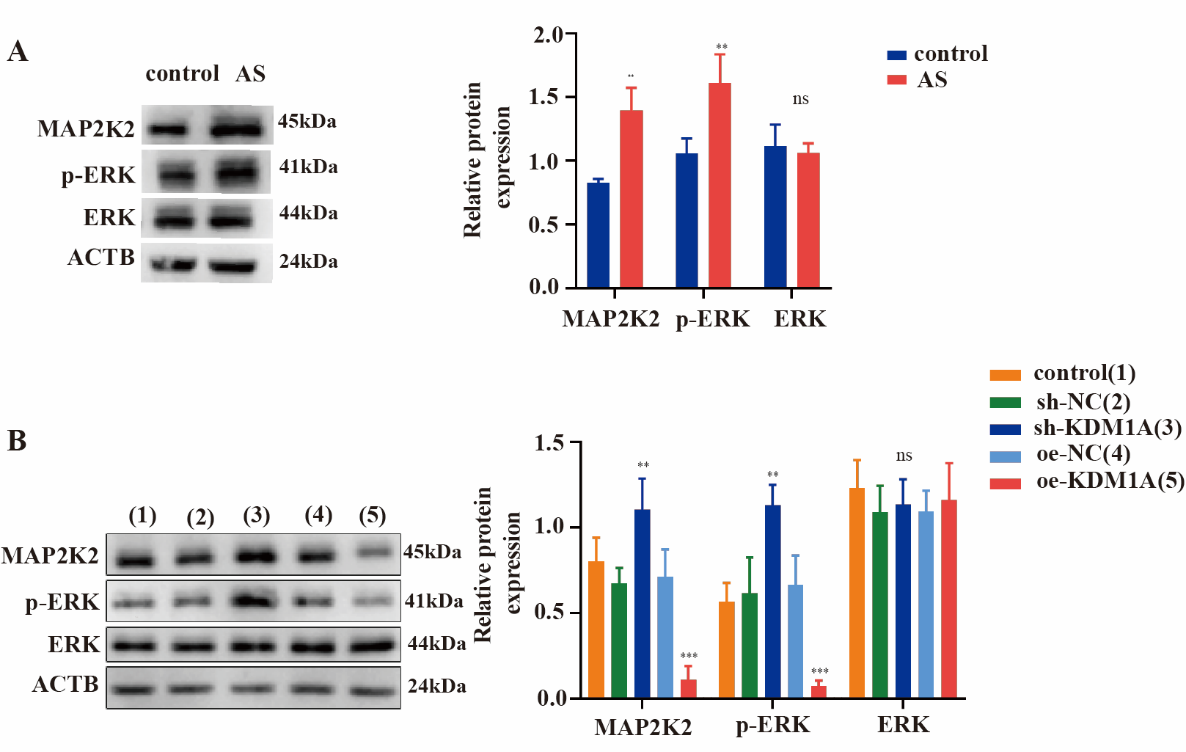
**

**Figure S4. Changes of MAPKs pathway in atherosclerosis**. (A) The relative levels of factors in MAPKs pathway in AS were assessed using western blotting. (B) The effects of KDM1A on MAPKs pathway were assessed using western blotting. ** *P* < 0.01 versus the control group; *** *P* < 0.001. All cellular experiments were repeated 3 times. Comparison among multiple groups was analyzed using Mann-Whitney test and one-way ANOVA.

**
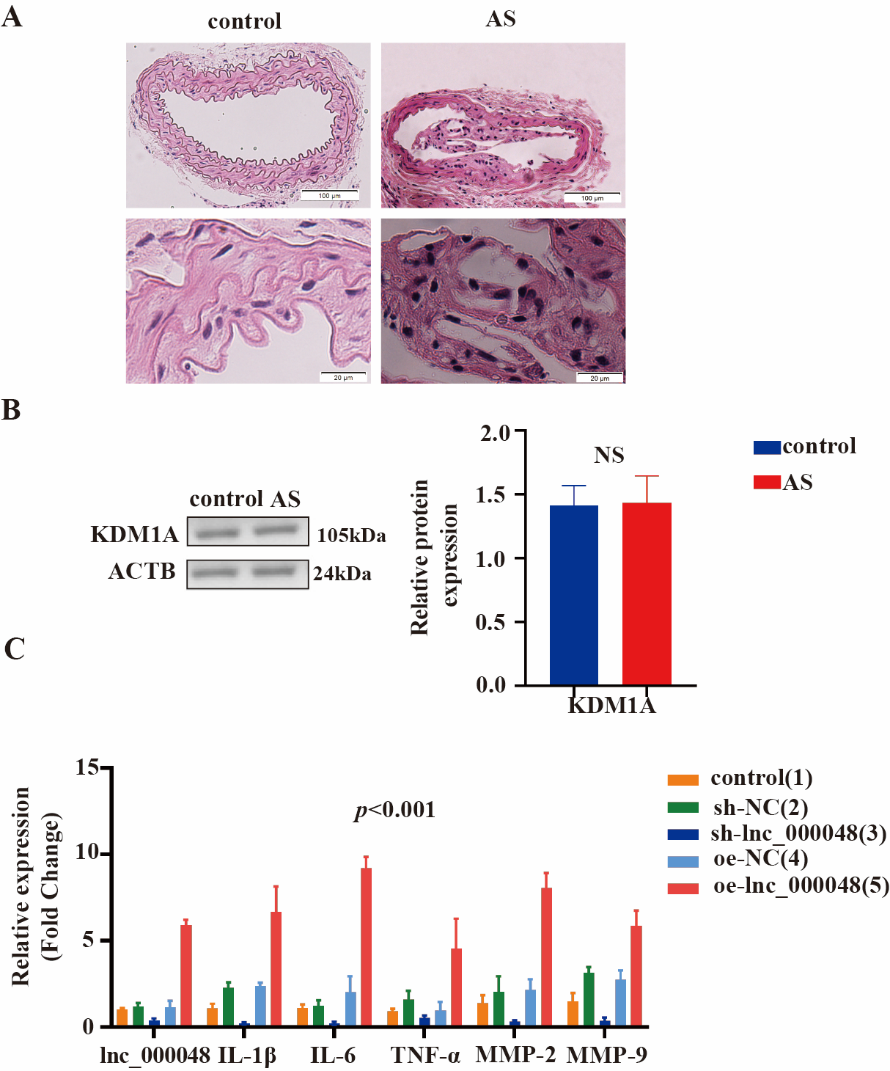
**

**Figure S5. The construction of atherosclerotic model and the role of inflamation of lnc_000048 in ApoE–/– mice.** (A) HE staining analysis of carotid plaque formation in ApoE-/- mice with atherosclerosis. All cellular experiments were repeated 3 times. (B) The level of KDM1A in plaque of atherosclerosis. (C)The effects of lnc_000048 on inflammatory cytokine and matrix metalloprotein gene expression assessed using qRT-PCR. Comparison among multiple groups was analyzed using one-way ANOVA.
